# Functionalized MoS_2_-erlotinib produces hyperthermia under NIR

**DOI:** 10.1186/s12951-019-0508-9

**Published:** 2019-06-19

**Authors:** Chen Zhang, Doudou Zhang, Jian Liu, Jie Wang, Yusheng Lu, Junxia Zheng, Bifei Li, Lee Jia

**Affiliations:** 1 Institute of Oceanography, Minjiang University, Wucheng Building, 5FL, No.200 Xiyuangong Road, Fuzhou, 350108 Fujian China; 2 Cancer Metastasis Alert and Prevention Center, and Pharmaceutical Photocatalysis of State Key Laboratory of Photocatalysis on Energy and Environment, College of Chemistry, Fujian Provincial Key Laboratory of Cancer Metastasis Chemoprevention and Chemotherapy, Fuzhou University, Sunlight Building, 6FL; Science Park, Xueyuan Road, University Town, Fuzhou, 350116 Fujian China

**Keywords:** MoS_2_, Drug delivery, Targeted, Synergistic chemo-photothermal therapy

## Abstract

**Background:**

Molybdenum disulfide (MoS_2_) has been widely explored for biomedical applications due to its brilliant photothermal conversion ability. In this paper, we report a novel multifunctional MoS_2_-based drug delivery system (MoS_2_-SS-HA). By decorating MoS_2_ nanosheets with hyaluronic acid (HA), these functionalized MoS_2_ nanosheets have been developed as a tumor-targeting chemotherapeutic nanocarrier for near-infrared (NIR) photothermal-triggered drug delivery, facilitating the combination of chemotherapy and photothermal therapy into one system for cancer therapy.

**Results:**

The nanocomposites (MoS_2_-SS-HA) generated a uniform diameter (ca. 125 nm), exhibited great biocompatibility as well as high stability in physiological solutions, and could be loaded with the insoluble anti-cancer drug erlotinib (Er). The release of Er was greatly accelerated under near infrared laser (NIR) irradiation, showing that the composites can be used as responsive systems, with Er release controllable through NIR irradiation. MTT assays and confocal imaging results showed that the MoS_2_-based nanoplatform could selectively target and kill CD44-positive lung cancer cells, especially drug resistant cells (A549 and H1975). In vivo tumor ablation studies prove a better synergistic therapeutic effect of the joint treatment, compared with either chemotherapy or photothermal therapy alone.

**Conclusion:**

The functionalized MoS_2_ nanoplatform developed in this work could be a potent system for targeted drug delivery and synergistic chemo-photothermal cancer therapy.

## Background

Cancer is one of the biggest challenges that threaten human health, among which lung and bronchus are the most common cause of cancer-related death [1–3]. Chemotherapy is still one of the frequently used therapeutic modalities for cancer treatment over the past decades, however chemotherapy suffers from several therapeutic bottlenecks, such as severe side effects, low solubility, and the tendency to induce drug resistance [4, 5]. Erlotinib (Er) is a clinical anticancer drugs working by selectively and reversibly inhibiting the epidermal growth factor receptor (EGFR) tyrosine kinase [6], thereby inhibiting downstream signaling pathways such as cell proliferation, metastasis, and angiogenesis [7]. Although Er has shown strong clinical therapeutic effect for lung cancer, the tumor therapeutic activity is still limited by above bottlenecks of chemotherapy, especially low solubility, instability and drug resistance. Therefore, it is necessary to develop an Er delivery system to overcome these defects and enhance its bioavailability.

Recently, a multitude of nanomaterials have been designed as they exhibit promising potential to overcome the limitations of chemotherapy drugs for cancer therapy applications, such as liposomes [8], carbon nanomaterials [9], silica nanoparticles [10], metal-based nanoparticles [11], polymeric nanoparticles [12–14] and quantum dots [15, 16] have been used in biomedical applications. Compared to these nanomaterials, two-dimensional (2D) nanomaterials possess exceptional chemical, optical, and electronic properties and are thus, being considered as novel therapeutic agents for biomedicine, especially for cancer treatment. There have been some particularly interesting reports that demonstrate encouraging potential of 2D nanomaterial theranostics in the pre-clinical area and targeted delivery of cancer therapeutics [12–17]. Since graphene oxide was applied, single layer 2D nanomaterials has drawn much attention in a wide range of areas because of their unique physical and chemical properties. Owing to these excellent properties, more effort has been paid to search for other similar 2D materials [17, 18]. MoS_2_ nanosheets, as a kind of transition metal dichalcogenides (TMDCs), displayed huge potential applications for nanoelectronic [19–21], transistors [22–24], energy storage devices [25, 26] and catalysis [27–29]. Past several years, a few groups have explored the promising application of single-layer MoS_2_ sheets in the biomedical field [30, 31]. Photothermal therapy (PTT) as a non-invasive therapeutic approach triggered by light, can transfer optical energy into heat, resulting in the thermal ablation of cancer cells [32, 33]. As a new type of 2D TMDCs, MoS_2_ has exhibited its intrinsic high NIR absorbance as well as outstanding photothermal conversion efficiency, which indicated that MoS_2_ could be used as a photothermal agent (PTA) for PTT [31, 34–36]. Besides, as a NIR photothermal delivery system, MoS_2_ has been reported that could stimulate the drug release triggered by NIR irradiation [37, 38]. Hence, the MoS_2_-nanosheets could be used to form a NIR-triggered drug delivery system because of the larger surface area and amazing photothermic. However, MoS_2_ nanosheets rapidly aggregate in physiological solution, which hampers the application of MoS_2_ nanosheets in the medical field. Therefore, the surface modification of single-layer MoS_2_ nanosheet remains a tremendous challenge for the application of the MoS_2_ nanosheets in biomedicine. Chou et al. showed that the thiolated molecules could be attached to the MoS_2_ nanosheets at the defect sites resulted from chemical exfoliation process and reported that the modified single-layer MoS_2_ sheets show outstanding biocompatibility and high absorbance when under the irradiation of NIR laser [34, 39]. The novel approach for the surface modification of single-layer MoS_2_ nanosheet is urgently needed.

Hyaluronic acid (HA), as a water-soluble biomacromolecule, has great biocompatibility and biodegradability [40, 41], Besides, HA is also a natural ligand for CD44 that is often overexpressed by various cancer cells, especially in drug-resistance cancer cells, and has been widely used in active targeting treatment of lung cancer [42–44]. MoS_2_ nanosheet modified with HA not only enhances its stability in physiological solution, but also could specifically combine with the drug-resistant tumor cells which overexpress CD44 [45]. Hence, this study was designed to demonstrate that Er-loaded MoS_2_ modified with HA could be engineered as a photothermal-triggered drug delivery system to specially target the CD44-overexpressing cancer cells, deliver non-water-soluble drug Er into cells, produce NIR-mediated hyperthermia, stimulate drug release triggered by photothermic, resulting in a synergistic cancer therapeutic effect in vitro and in vivo.

## Materials and methods

### Materials

Molybdenum sulfide (MoS_2_, 99%) was purchased from Sigma Aldrich (CA, USA). Cystamine dihydrochloride was bought from Bide Pharmatech Co., Ltd (Shanghai, China). Hyaluronic acid (MW = 35 kDa) was purchased from Shandong Freda biological Technology Co., Ltd (Jinan, China). Erlotinib (Er, 99%) was provided by Dalian Meilun biological Technology Co., Ltd (Dalian, China). All reagents related to cell culture were purchased from Hyclone (Logan, UT, USA). Other reagents were obtained from J&K Scientific Ltd (Shanghai, China). All the chemicals were used as received without further purification.

### Preparation of single-layer MoS_2_-SS-HA nanosheets

#### Synthesis of MoS_2_ nanosheets

MoS_2_ nanosheets were synthesized by using the Morrison method [46]. In brief, 0.5 g MoS_2_ flakes were stirred with a solution of *n*-butyllithium in hexane (0.5 mL; 1.6 M) under N_2_ atmosphere for 48 h, performed in a nitrogen glove box. After intercalation by lithium, the sample was centrifuged and washed repeatedly with hexane to remove leftover lithium and additional organic residues. Intercalated MoS_2_ solution was then dislodged from the glove box and instantly ultra-sonicated in water for 1 h to obtain exfoliated MoS_2_, besides, the unexfoliated MoS_2_ and excess LiOH were removed by centrifugation at 3000 rpm. The supernatant containing exfoliated MoS_2_ was dialyzed against water for 48 h to remove excess impurities and the finally obtained MoS_2_ nanosheets aqueous solution was stored at 4 °C for future use.

#### Preparation of targeted nanocomposite

200 mg HA was first dissolved in 20 mL deionized water, then 38 mg ethylene dichloride (EDC) and 46 mg *N*-hydroxysuccinimide (NHS) were added under stirring. Afterwards cystamine dihydrochloride (1.2 g) was added to the mixture and stirred overnight. The product, denoted HA-SS, was obtained after dialyzing against water with a cellulose membrane (MWCO: 1 kDa) for 24 h. 5 mg HA-SS was added to a dispersion of MoS_2_ (0.25 mg/mL, 4 mL water) and then ultrasonicated for 30 min. After stirring overnight, the resultant product (MoS_2_-SS-HA) was dialyzed with water against a cellulose membrane (MWCO: 100 kDa). The obtained MoS_2_-SS-HA nanosheets were stored at 4 °C until use.

### Erlotinib loading and releasing

#### Erlotinib loading

Erlotinib (Er) loading onto MoS_2_-SS-HA was applied according to the following protocol. In brief, MoS_2_-SS-HA nanosheets watery solution (0.5 mg/mL) was mixed with different concentrations of Er which were dissolved in DMSO solution and stirring the mixture for 24 h (pH = 7.0). Excess Er was removed by centrifugation at 4000 rpm for 20 min. The supernatant was filtered (0.45 μm) to remove the remaining undissolved Er. The obtained solution was centrifuged for 5 times by ultrafiltration (10 kDa MWCO) to remove the dissolved excess Er. Loading amount of Er was detected using UV–vis spectra absorbance peak at 343 nm.

#### Erlotinib releasing

The methods were similar to what we described previous [47–49]. Briefly, 2.5 mg MoS_2_-SS-HA-Er nanosheets were suspended in PBS buffer (5 mL, pH = 7.4) or acetate buffer (5 mL, pH = 5.6) and placed into dialysis bags (10 kDa MWCO). After 1 h, the samples were irradiated by 808 nm NIR laser with a power density of 1 W/cm^2^ for 20 min. The release assay was performed on a shaking bed at 37 °C at a speed of 100 rpm. Each of 0.5 mL dialysate was collected at designed time points and replaced with the same volume of fresh buffer solution. The released amount of Er from MoS_2_-SS-HA-Er was quantified by UV–Vis spectroscopy. The accumulative amount of Er released from the composites was calculated as follows:

$$ {\text{Accumulative release of Er }}\left( \% \right)\, = \,\left( {{\text{Total amount of Er}} - {\text{amount of free Er}}} \right)/{\text{Total amount of Er}}\, \times \, 100\% . $$

### Materials characterization

The morphology of MoS_2_ was measured using transmission electron microscopy (TEM; JEM-2100, JEOL). The thickness and size of the MoS_2_ particles were determined with a 5500 atomic force microscope (AFM; Agilent). The zeta potential was quantified with a ZS90 Zetasizer instrument (Malvern Instruments). Dynamic light scattering (DLS) was performed with static light scattering instrument (BI-200SM, Brookhaven Instruments). UV–vis spectra were obtained on a UV3600 instrument (Shimadzu Corporation). Fourier transform infrared (FT-IR) spectroscopy was recorded on a Vetex70 (Bruker Corp., Germany). The photothermal properties of the composite were examined using a laser device (Shanghai Xilong Optoelectronics Technology Co. Ltd.) at a wavelength of 808 nm.

### Cell culture

A549 (EGFR wide-type, erlotinib-initially resistant), H1975 (EGFR-mutated subtype, L858R/T790M double mutations, erlotinib-acquired resistant), PC-9 (EGFR-mutated subtype, exon 19 deletion, erlotinib-sensitive) and HELF cells were purchased from the Cell Resource Center of Shanghai Institute for Biological Sciences (Chinese Academy of Sciences, Shanghai, China). These cells were cultured in the recommended medium at 37 °C within 5% CO_2_ atmosphere.

### Biocompatibility of MoS_2_-SS-HA in vitro and in vivo

#### Cytotoxicity assays in vitro

The methods were described in our previous study [48]. In brief, cells were seeded in 96-well cell culture plates at a density of 8 × 10^3^ cells/well and incubated with MoS_2_-SS-HA at different concentrations (12.5, 25, 50, 100, 200 μg/mL). After 24 h incubation, the cells viabilities were measured by MTT assay.

#### Hemolysis assays of MoS_2_-SS-HA in vivo

Hemolysis assay were performed as following: 1 mL blood obtained from rat (Wistar, female, 4–6 weeks old, purchased from Fuzhou Wushi Animal Center) was treated with ethylene diamine tetracetic acid. Then the blood was centrifuged at 1000 rpm for 10 min and removed the upper serum carefully. The lower red blood cells were diluted 30 times with PBS. Next, 0.3 mL of diluted red blood cells was mixed with (i) 0.9 mL of PBS as a negative control, (ii) 0.9 mL of water as a positive control, (iii) 0.9 mL of MoS_2_-SS-HA dispersions at different concentrations (50, 100, 200, 400, 800 μg/mL). Eventually, all the mixtures were kept shaking at 100 rpm for 2 h and then centrifuged at 12,000 rpm for 10 min. The absorbance of supernatants was detected at 541 nm by UV–vis spectrophotometry. Hemolysis percentage (%) = (A sample-A negative)/(A positive-A negative) × 100%.

#### Biocompatibility of assays in vivo

Biocompatibility of MoS_2_-SS-HA was carried out in vivo in accordance with the protocol approved by Institutional Animal Care and Use Committee. BALB/c nude female mice (5–7 weeks old) were purchased from Fuzhou Wushi Animal Center and maintained in cages in a SPF-grade animal room with access to food and water ad libitum. After 1 week of adaptation feeding, nude mice were randomly divided into two groups (n = 5), then nude mice were treated with 100 μL of (i) saline, (ii) MoS_2_-SS-HA in saline (2.0 mg/kg), via tail vein injection. The injection was performed every 2 days. After 3 weeks, all mice were sacrificed and the major organs (heart, liver, spleen, lung and kidney) were obtained and were stained with hematoxylin–eosin (H&E) to observe histopathological changes.

### In vitro cellular uptake

Intracellular uptake of the FITC-loading MoS_2_-SS-HA nanosheets was judged by confocal laser scanning microscopy (CLSM, Leica TCS SP8, IL, USA) and flow cytometry (BD FACSAriaIII, BD Bioscience). As a fluorescent probe, the FITC was loaded on the MoS_2_-SS-HA nanosheets in the same protocol as Er (shown in 2.3.1 Er loading procedure). Cells were seeded in 6-well plates (1 × 10^5^ cells/well) and cultured for 24 h. The media was aspirated and 2 mL of fresh DMEM containing 0 or 5 mg/mL HA was added. 2 h later, cells were washed twice with PBS and incubated with MoS_2_-SS-HA-FITC (FITC = 5 μg/mL). The cells in all the groups were cultured for another 2 h and then washed and fixed with glutaraldehyde for 30 min. The cell nuclei were stained with DAPI and detected by CLSM and flow cytometry.

### Cytotoxicity of MoS_2_-SS-HA-Er in vitro

The cytotoxicity of MoS_2_-SS-HA-Er against lung cancer cells (A549, H1975, PC-9) was assessed by MTT assay. Briefly, cells were seeded in 96-well plates and cultured overnight. Then media was aspirated and a solution of Er (200 μL, [Er] = 1.25, 2.5, 5, 10, 20 μg/mL) were added to each well, and the plates incubated at 37 °C in a 5% CO_2_ atmosphere for 24 h. The MTT reagent (10 μL, 5 mg/mL) was then added, followed by incubation at 37 °C in a 5% CO_2_ atmosphere for 4 h. The supernatant was then carefully removed and the MTT-formazan produced by live cells solubilized in 150 mL of DMSO for 20 min. Finally, the absorbance at 490 nm was measured using a microplate reader (MULTSIKAN MK3, Thermo Fisher). Cell viability (%) was determined from the absorbance at 490 nm and normalized to a negative control wells containing untreated cells. Experiments were performed in triplicate.

A second set of experiments was performed to assess the potential for photochemo-therapies. Cells were cultured in a 96-well plate at 1 × 10^4^ cells per well (200 μL of cell suspension per well) for 24 h, and then co-cultured with Er or MoS_2_-SS-HA-Er. Cells were divided into 4 treatment groups at Er concentrations ranging from 1.25 to 20 μg/mL as follows: (i) Saline + NIR; (ii) Free Er + NIR; (iii) MoS_2_-SS-HA-Er + NIR (synergistic therapy); (iv). MoS_2_-SS-HA + NIR. After incubation for 12 h, the cells were washed with 100 μL PBS and 100 μL culture medium was then added to the wells. The cells were irradiated with an 808 nm laser at different power densities (0.5 W, 0.8 W, 1.2 W) for 20 min, before the cells were cultured for a further 24 h and an MTT assay used to measure cell viability.

### Detection of cell apoptosis and cell cycle

To evaluate the therapeutic efficacy of the nanosheets, cell cycle and apoptosis were measured by using flow cytometry according to following protocol. Briefly, cells were seeded at a density of 3 × 10^5^ cells/well in the six-well plates. After 24 h, the cells were separated into five groups, as following: control, Er, MoS_2_-SS-HA + NIR, MoS_2_-SS-HA-Er and MoS_2_-SS-HA-Er + NIR ([Er] = 10 μg/mL). Besides, groups of MoS_2_-SS-HA + NIR and MoS_2_-SS-HA-Er + NIR were exposed to 808 nm-NIR laser with a power density of 1.2 W/cm^2^ for 20 min. Finally, the cells were collected and detected by flow cytometry according to the instruction of Apoptosis Assay Kits (Keygen BioTech, Nanjing, China). For cell cycle assay, the cells were collected and washed thrice with ice-cold PBS. Then the cells were fixed with cold 70% ethanol for 24 h at 4 °C. Subsequently, the cells were centrifuged and washed twice with PBS. Ultimately, the staining solution consisting of 1% (v/v) Triton X-100, 0.01% RNase, and 0.05% PI was added to the cells and stained for 30 min in darkness before detection by flow cytometry.

### Antitumor effects of MoS_2_-SS-HA-Er in vivo

All animal experiments were carried out in accordance with the protocol approved by Institutional Animal Care and Use Committee. BALB/c nude female mice (5–7 weeks old) were purchased from Fuzhou Wushi Animal Center and maintained in cages in a SPF-grade animal room with access to food and water ad libitum. A549 cells (1 × 10^6^ cells/well) were suspended in 100 μL PBS were subcutaneously injected into the left fore limb of each nude mouse to form the tumor model. The tumor volume was measured using vernier caliper and calculated as V = (length × width^2^)/2. The nude mice were randomly divided into six groups (n = 5) when the tumor volume reached up to 90 mm^3^. Afterward, each group of the tumor-bearing nude mice was treated with 100 μL of (i) saline, (ii) Er (2.0 mg/kg), (iii, iv) MoS_2_-SS-HA in saline (2.0 mg/kg), and (v, vi) MoS_2_-SS-HA-Er (2.0 mg/kg) in saline, respectively, via tail vein injection. After 8 h, the mice of group (iv) and (vi) were treated with 808 nm-NIR laser light (0.5 W/cm^2^) for 10 min. The infrared ray (IR) images of tumors were observed with an infrared thermal camera (InfReC R500EX, Tokyo, Japan), showing the real-time temperature changes with irradiation. During the treatment, the tumors volume and the weight of mice were recorded every 3 days.

### Statistical analysis

Statistical analysis was performed with GraphPad Prism 5.0 (GraphPad software, San Diego, CA). In general, for two experimental comparisons, a two-tailed unpaired Student’s *t* test was used unless otherwise indicated. For multiple comparisons, one-way ANOVAs were applied. When cells were used for experiments, three replicates per treatment were chosen as an initial sample size. All n values defined in the legends refer to biological replicates. Data were assessed as mean ± SD. Statistical significance was set at *p < 0.05 and high significance was set at **p < 0.01.

## Result and discussion

### Preparation and characterization of nanocomposites

**Fig. 1:**
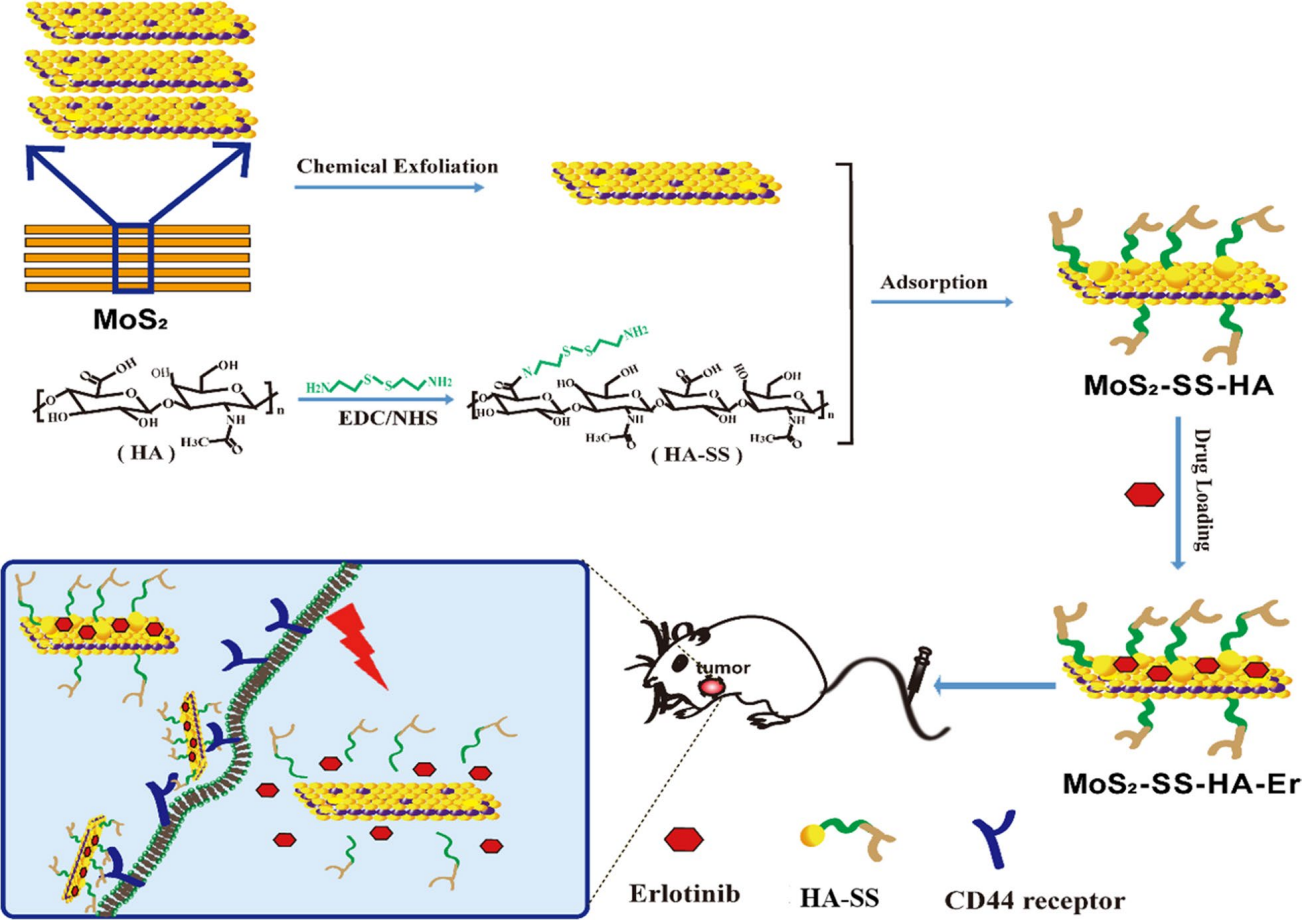
Schematic illustration depicting the construction of MoS_2_-SS-HA-based nanocomposites for synergistic chemo-photothermal therapy. Chemical exfoliation of MoS_2_ flakes to nanosheets and synthesis of HA-SS-NH_2_ via EDC/NHS reaction. Subsequently, the MoS_2_ nanosheets are functionalized with HA and loading Er at the defect sites to obtain MoS_2_-SS-HA-Er nanosheets, which could target tumor site mediated by CD44 receptor, and produce the hyperthermia upon NIR irradiation

Figure 1 demonstrates the preparation of HA-modified MoS_2_ nanosheets as a multifunctional nano-platform for targeted and photothermal-responsive therapy of tumor. First, the single-layer MoS_2_ nanosheet was prepared following the method of chemical exfoliation [50]. Then HA was decorated on the surface of single-layer MoS_2_ nanosheet with the cystamine dihydrochloride to form MoS_2_-SS-HA. Finally, Er was loaded on the surface of MoS_2_-SS-HA to obtain MoS_2_-SS-HA-Er. This nanosheets integrates the multifunction of insoluble drug targeted delivery, PTT and controlled drug release.

**Fig. 2:**
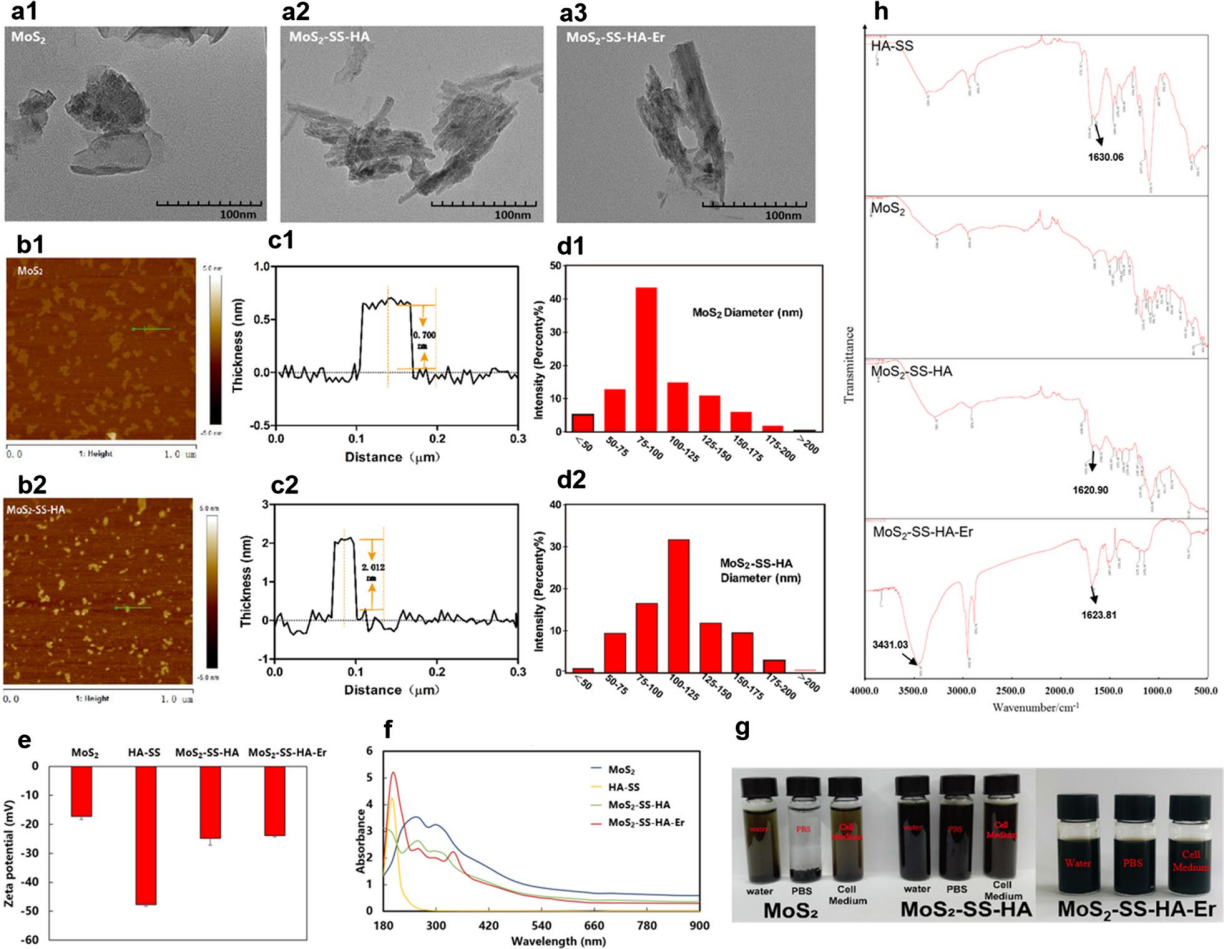
Physicochemical characterization. TEM (**a1**, **a2**, **a3**) images of MoS_2_, MoS_2_-SS-HA and MoS_2_-SS-HA-Er. Images (**b1**, **b2**) and the corresponding AFM height profile (**c1**, **c2**) of MoS_2_ and MoS_2_-SS-HA by atomic force microscopy (AFM) (**d1**, **d2**) The size distribution of MoS_2_ (75 nm) and MoS_2_-SS-HA (125 nm) nanosheets by dynamic laser light scattering (DLS). **e** Zeta potential of MoS_2_, HA-SS, MoS_2_-SS-HA and MoS_2_-SS-HA-Er. **f** UV–vis absorption spectra of MoS_2_, HA-SS, MoS_2_-SS-HA and MoS_2_-SS-HA-Er. **g** Photographs of MoS_2_, MoS_2_-SS-HA and MoS_2_-SS-HA-Er nanosheets in water, PBS and cell medium for 1 month. The dispersibility of MoS_2_ nanosheets and the solubility of Er were improved after modified with HA. **h** FT-IR spectra for MoS_2_, HA-SS, MoS_2_-SS-HA and MoS_2_-SS-HA-Er, and the band at 3431.03 cm^−1^ correspond to the characteristic vibration of the –C≡H bond due to the coupling vibration of Er

The typical morphologies, structures, and dispersity of MoS_2_, MoS_2_-SS-HA and MoS_2_-SS-HA-Er were analyzed by transmission electron microscopy (TEM). In Fig. 2a, it is shown that MoS_2_ nanosheets have a well-defined laminar morphology with a size of around 70 nm, while MoS_2_-SS-HA and MoS_2_-SS-HA-Er have a wrinkled sheets and larger-size (125 nm), which are usually observed for modification of nanosheets. The morphology of prepared MoS_2_-SS-HA was characterized by AFM images, which shows the material to consist of exfoliated sheets (Fig. 2b), the results are consist with the literature of MoS_2_ [51]. The AFM data in Fig. 2c, show that the thickness of MoS_2_ nanosheets is approximately 0.7 nm, indicating that the acquired nanosheet was single layer [52]. The height of nanosheets increased to about 2 nm after HA coating, revealing the successful coating of HA on the surface of MoS_2_ nanosheets. The hydrodynamic diameters of the nanocomposites were quantified by DLS, and the results are shown in Fig. 2d. The hydrodynamic diameters of MoS_2_ and MoS_2_-SS-HA nanosheets detected by DLS were 75 nm and 125 nm, respectively, which is appropriate for use as cell-targeted drug delivery system.

Zeta potential of MoS_2_, MoS_2_-SS-HA and MoS_2_-SS-HA-Er were shown in Fig. 2e. The zeta potential of MoS_2_-SS-HA is − 24.8 mV (HA-SS is − 17.3 mV and MoS_2_ is − 47.7 mV), there is no significant difference between MoS_2_-SS-HA and MoS_2_-SS-HA-Er in accordance with literature [53]. UV–vis spectroscopy further validated the successful synthesis of MoS_2_-SS-HA-Er (Fig. 2f). The characteristic peak of MoS_2_ is between 260 to 300 nm. The characteristic peak of HA is around 192 nm, similar to literatures [54, 55]. The characteristic peak of Er is around 335 nm in accordance with references [56, 57]. MoS_2_-SS-HA-Er showed both characteristic peaks of HA and Er.

As expected, physiological stability of MoS_2_ nanosheets was improved after modified with HA. In addition, the MoS_2_ nanosheets generated obvious aggregation among in water, PBS and cell medium within 1 h, while no obvious aggregation was observed in water and other physiological solutions for 1 month after decorated with HA, which demonstrated the good dispersibility of MoS_2_-SS-HA and MoS_2_-SS-HA-Er in physiological solutions (Fig. 2g). To confirm the combination of Er and MoS_2_-SS-HA sheets, Fourier transform infrared (FT-IR) spectroscopy was conducted. As shown in Fig. 2h, the amide group (–CO–NH–) of HA appears as a characteristic band at about 1600 cm^−1^, and the band at 3431.03 cm^−1^ correspond to the characteristic vibration of the –C≡H bond due to the coupling vibration of Er.

### Photothermal activity

**Fig. 3:**
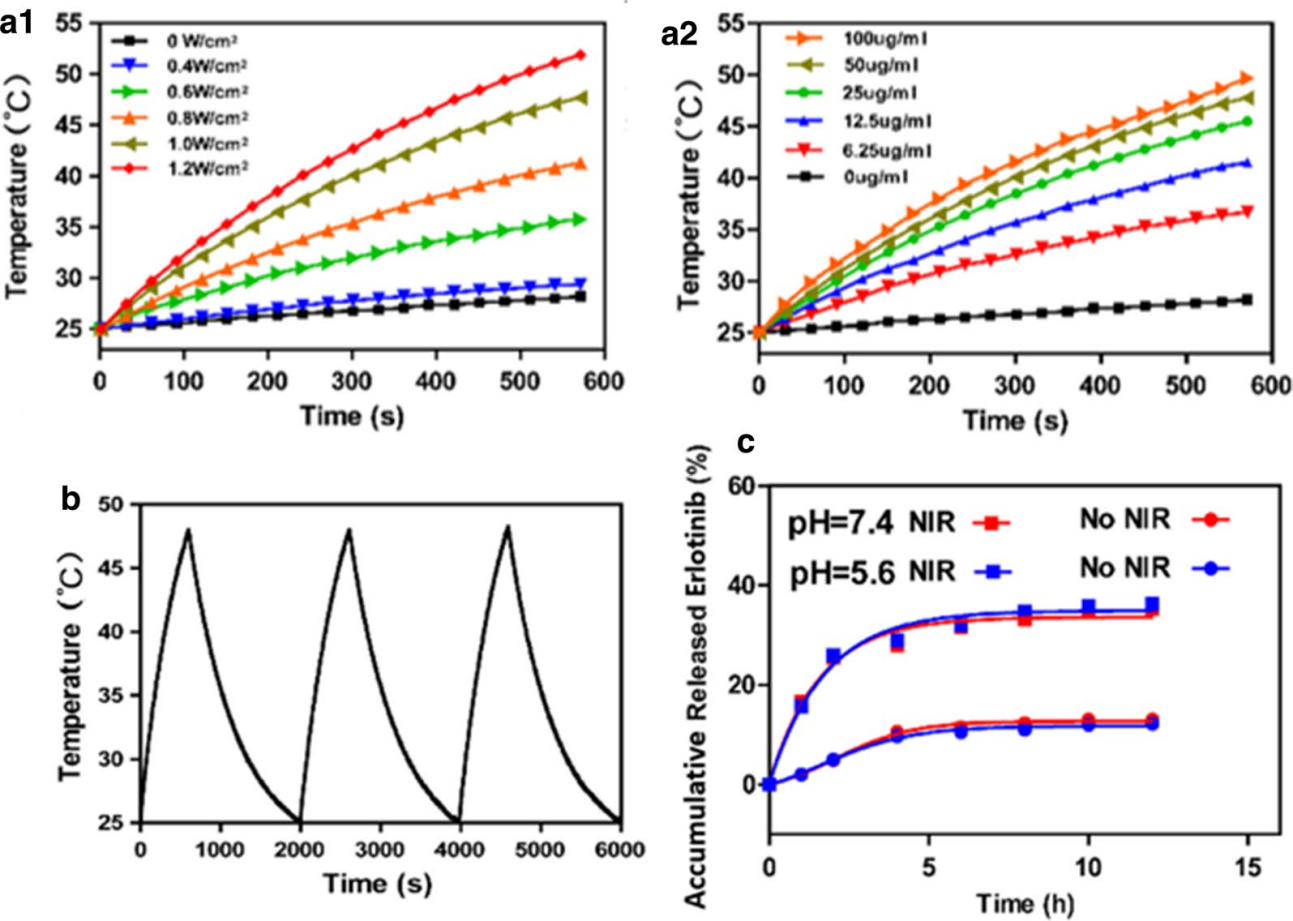
Photothermal effect, drug loading and releasing capacity of MoS_2_-SS-HA nanosheets. **a1** Heating curves of a 0.5 μg/mL suspension of MoS_2_-SS-HA under different laser power densities; **a2** Heating curves of different concentrations of MoS_2_-SS-HA under laser power at 1.0 W/cm^2^. **b** A plot showing the response of MoS_2_-SS-HA over three on–off cycles (0.5 μg/mL suspension, 1.0 W/cm^2^ laser density). **c** Er release profiles of MoS_2_-SS-HA-Er at different pH (pH = 5.6 or 7.4) with and without 808 nm laser irradiation (1.0 W/cm^2^)

It is well known that MoS_2_ nanocomposites have good photothermal conversion efficiency [58, 59], and hence the photothermal activity of MoS_2_-SS-HA was explored in a series of tests. In Fig. 3a1, the photothermal heating effect of a 50 μg/mL suspension of the MoS_2_-SS-HA composite under irradiation by different laser power is depicted. A laser power intensity dependent photothermal effect was observed, as would be expected. When the laser power was 1.2 W/cm^2^ the temperature reached 50 °C within 500 s. The photothermal effect is found to be concentration-dependent under irradiation at 0.5 W/cm^2^ (Fig. 3a2). These results indicate the suitability of MoS_2_-SS-HA for photothermal therapy and potential for the thermal ablation of tumors. After three on–off cycles of irradiation (Fig. 3b), the temperature response of the MoS_2_-SS-HA the laser power was 1.2 W/cm^2^ the temperature reached 50 °C within 500 s. The photothermal effect is found to be concentration-dependent under irradiation at 0.5 W/cm^2^ (Fig. 3c). These results indicate the suitability of MoS_2_-SS-HA for photothermal therapy and potent suspension remains largely constant. This suggests that MoS_2_-SS-HA has excellent photothermal stability.

### In vitro drug release

MoS_2_-SS-HA nanosheet has ultrahigh surface area and a water-soluble biomacromolecule, therefore could be used as a drug carrier [30, 60]. We loaded an insoluble chemotherapeutic drug, erlotinib (Er), on the MoS_2_-SS-HA nanosheets to obtain the MoS_2_-SS-HA-Er. It was shown in Fig. 2f that Er had been loaded on the surface of MoS_2_-SS-HA nanosheets by UV–vis spectra.

Drug release from the nanosheets was affected by many experimental factors [61, 62], the most commonly studied are pH and NIR irradiation. The in vitro release behavior of MoS_2_-SS-HA-Er was investigated in different pH media (5.6 and 7.4) with and without laser irradiation (Fig. 3c). The release of Er was almost no difference between pH 5.6 and pH 7.4. At a given pH, there is greater Er release with laser irradiation than without. This indicates that NIR light-triggered photothermal heating could promote the release of Er and accelerate the death of cancerous cells. For example, the cumulative release of Er after 12 h at pH 7.4 with laser irradiation (33.3%) was much greater than that at pH 7.4 without laser irradiation (8.9%).

### Biocompatibility of MoS_2_-SS-HA in vitro and in vivo

**Fig. 4:**
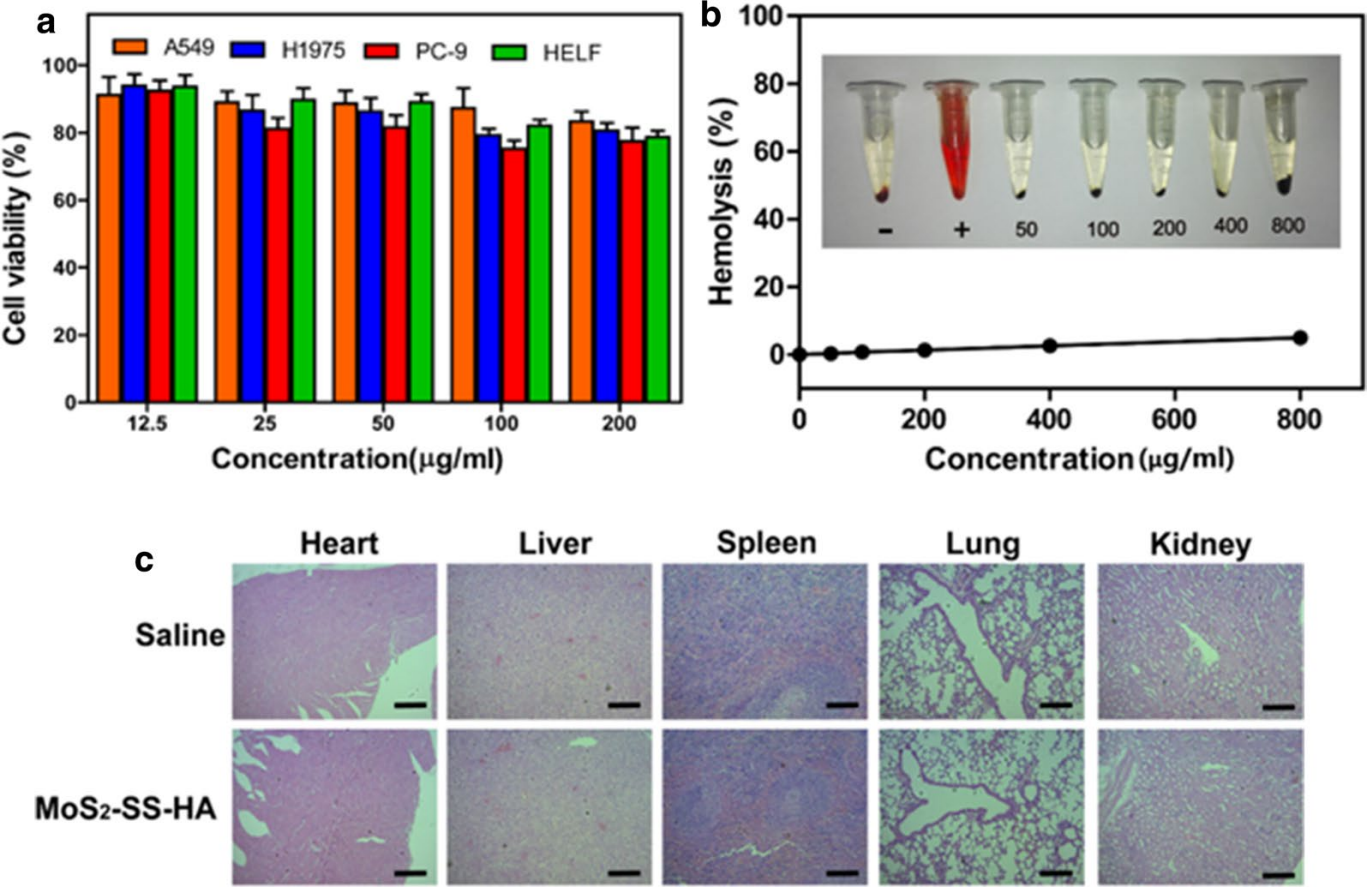
The biocompatibility of MoS_2_-SS-HA. **a** Cell viability of A549, H1975, PC-9 and HELF cells incubated with MoS_2_-SS-HA with different concentrations for 24 h. **b** Hemolytic percentage of red blood cells (RBCs) incubated with MoS_2_-SS-HA at different concentrations for 2 h (“−” means PBS group, “+” means water group). Inset: picture for direct observation hemolysis of MoS_2_-SS-HA, showing the good biocompatibility of MoS_2_-SS-HA nanosheets. **c** H&E staining of major organs from control (saline treated) and MoS_2_-SS-HA group (via tail vein injection every 2 days for 3 weeks). No significant organ toxicity was observed, which further proved the bio-safety of MoS_2_-SS-HA for biomedical applications. Scale bar: 100 μm

Biocompatibility is an essential concern when it comes to the development of nanomaterials for biomedical application. An ideal drug delivery platform must be biocompatible, non-toxic and not be associated with incidental adverse effects [62, 63]. Herein, before conducting further experiments in vitro and in vivo, the cytotoxicity of MoS_2_-SS-HA nanosheets against cells was measured by MTT assay. As shown in Fig. 4a, the viability of all cells remained over 80% at high concentration of MoS_2_-SS-HA, indicating the ultralow cytotoxicity of MoS_2_-SS-HA against lung cancer cells (PC-9, A549 and H1975) and HELF cells after 24 h incubation. In addition, hemolysis assay results indicated that negligible hemolysis was observed in MoS_2_-SS-HA groups, even the concentration reached to 800 μg/mL, demonstrating that the MoS_2_-SS-HA nanosheets has excellent blood compatibility (Fig. 4b). Further, as shown in Fig. 4c, H&E staining results revealed little histopathological changes between saline group and MoS_2_-SS-HA group. Hence, good compatibility of the MoS_2_-SS-HA nanosheets both in vitro and in vivo indicate the great application potential in the cancer treatment.

### Cellular uptake in vitro

**Fig. 5:**
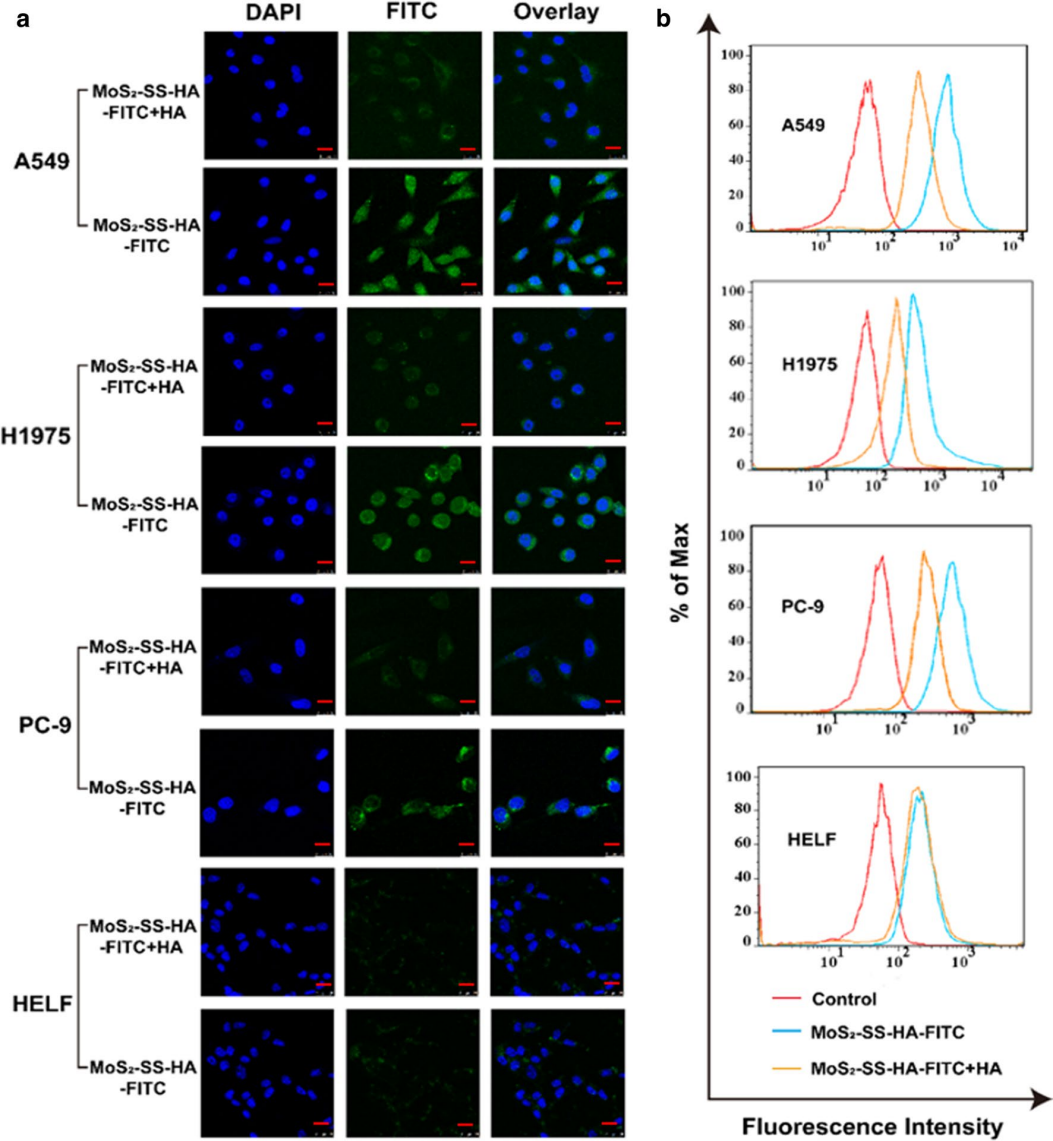
The intracellular uptake of MoS_2_-SS-HA using FITC as a fluorescent probe. Confocal fluorescence images (**a**) and quantitative flow cytometric analyses (**b**) of A549, H1975, PC-9, and HELF cells which were incubated with MoS_2_-SS-HA-FITC for 2 h (“+HA” means pretreat with 5 mg/mL HA for 2 h). Intensity of FITC fluorescence was obviously decreased with pretreatment of HA, demonstrating the HA-mediated intracellular uptake. A549 (EGFR wide-type, erlotinib-initially resistant), H1975 (EGFR-mutated subtype, L858R/T790M double mutations, erlotinib-acquired resistant) and PC-9 (EGFR-mutated subtype, exon 19 deletion, erlotinib-sensitive). The bar represents 25 μm

HA-coated nanoparticles could target the tumor site [64]. The targeting ability of the nanocomposites was investigated by CLSM. A549, H1975 and PC-9 which are HA-receptor positive, while HELF is HA-receptor negative, were cultured with MoS_2_-SS-HA-FITC and MoS_2_-SS-HA-FITC + HA, as shown by the coincident presence of DAPI (blue) and FITC (green) fluorescence (Fig. 5a). Relatively little MoS_2_-SS-HA-FITC is taken up by HA-receptor negative cell line HELF, and a little amount is taken up by erlotinib sensitive cell line PC-9. In contrast, the uptake of MoS_2_-SS-HA-FITC by erlotinib resistant cell line A549 and H1975 was similar, both exhibiting much greater FITC fluorescence than HELF. This arises because of the lack of an HA receptor on HELF. In order to clarify the cellular uptake mediated by HA receptor, additional HA was added to block CD44 (HA receptor). After HA added, negligible FITC green fluorescence was detected in all cell lines. The quantitative flow cytometry data (Fig. 5b) were consistent with the confocal imaging results, which further indicated MoS_2_-SS-HA is an excellent CD44-mediated cancer cell targeting drug delivery platform [43, 44, 65–68].

### Cytotoxicity of MoS_2_-SS-HA-Er in vitro

**Fig. 6:**
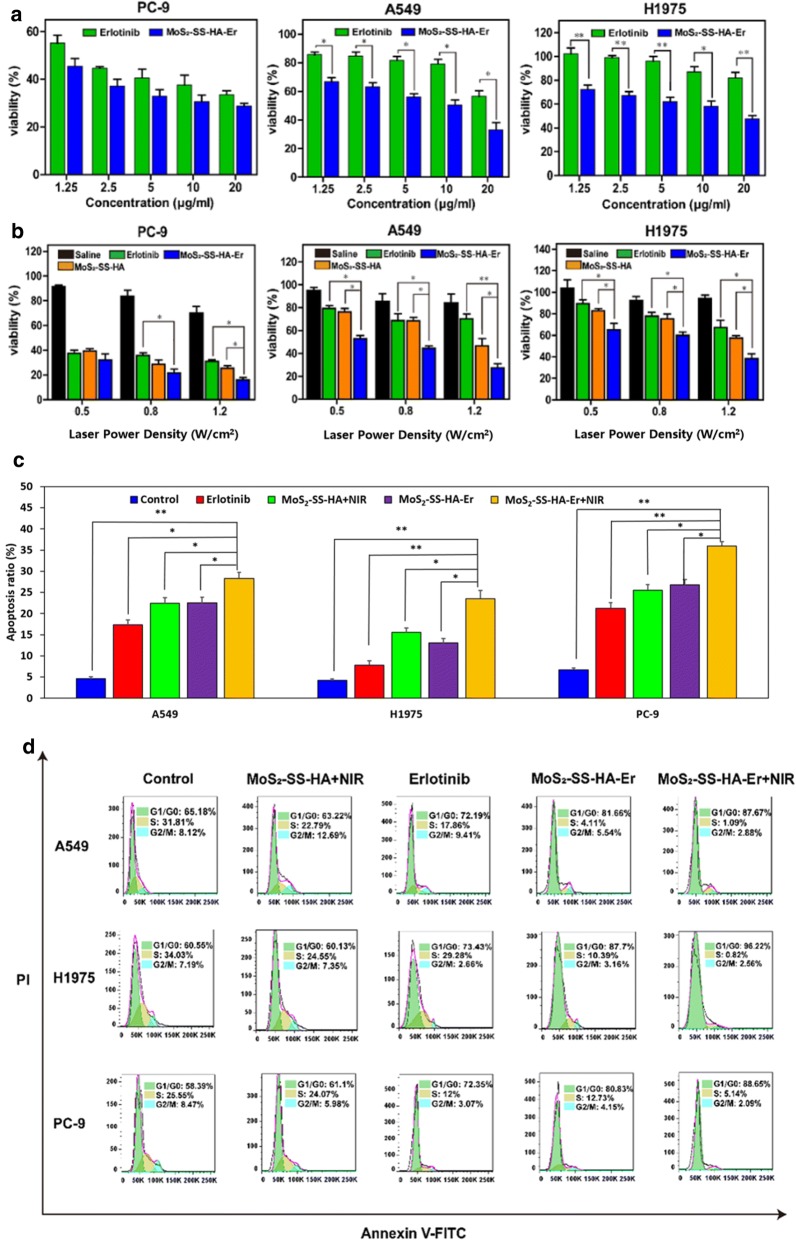
The biological effect of MoS_2_-SS-HA-Er with NIR in vitro. **a** Cytotoxicity of A549, H1975 and PC-9 cells treated with Er or MoS_2_-SS-HA-Er at different concentrations. **b** Cytotoxicity of A549, H1975 and PC-9 cells treated with Er or MoS_2_-SS-HA-Er ([Er] = 10 μg/mL) at different 808 nm NIR irradiation laser power densities. The apoptosis ratio (**c**) and the cell cycle (**d**) were analyzed by flow cytometry treated with saline, free Er, MoS_2_-SS-HA + NIR, MoS_2_-SS-HA-Er and MoS_2_-SS-HA-Er + NIR ([Er] = 10 μg/mL, power = 1.2 W/cm^2^). Significant differences between groups are labeled with *p < 0.05 and **p < 0.01

The therapeutic efficacy of free Er and MoS_2_-SS-HA-Er against cancer cells was investigated by MTT assays after incubation with PC-9, A549 and H1975 (Fig. 6a). The cell viability of erlotinib-sensitive cell line PC-9 was below 40% with treatment of free Er (20 μg/mL), while the viability of erlotinib-resistant cell line A549 and H1975 is above 60% and 80%, respectively, with treatment at high concentrations of 20 μg/mL free Er, indicating free Er revealed poor therapeutic efficiency against erlotinib-resistant cell lines. The excitement is the viability of erlotinib-resistant cell line (A549 and H1975) decreased below 40% with treatment of MoS_2_-SS-HA-Er at a concentration of 20 μg/mL. Considering the cell viability at equivalent doses of Er, in the case of A549 and H1975 cells the MoS_2_-SS-HA-Er group has markedly lower viability than cells treated with the free drug, the reason may be that the nanosystem is embedded in lysomal vesicles by CD44 receptor-mediated endocytosis [69]. To further explore the cell-killing effect of MoS_2_-SS-HA-Er combined with hyperthermia, cells were incubated with saline (control), Er, MoS_2_-SS-HA and MoS_2_-SS-HA-Er at the same Er concentration ([Er] = 10 μg/mL) exposed to the various density of NIR laser irradiation (Fig. 6b). NIR irradiation alone was found to have no effect on cell viability: A549 and H1975 cells showed essentially identical cell viability (> 85%) when they were treated with laser irradiation at a density from 0.5 to 1.2 W/cm^2^. For all cell types, the viability of cells treated with MoS_2_-SS-HA-Er ([Er] = 10 μg/mL) and laser irradiation are lower than those receiving MoS_2_-SS-HA-Er alone, indicating the PTT effect of the MoS_2_ nanocomposites. The erlotinib-resistant A549 and H1975 cell viabilities in the combined therapy group are much lower than the monotherapy groups (chemotherapy or PTT), showing the synergistic benefits of simultaneous PTT and chemotherapy. This leads to enhanced chemotherapy. Given that the cytotoxic effect of MoS_2_-SS-HA-Er with or without laser irradiation was higher with A549 and H1975 cells than PC-9 cells, there is also the potential for selective killing of drug-resistance cancer cells. All these results confirm that the multifunctional drug delivery system constructed in this work is effective in killing tumor cells and promising in chemo-photothermal combined cancer therapy.

### Cell cycle and cell apoptosis

The result of cells apoptosis assay was illustrated in Fig. 6c. The apoptosis percentages of the cells incubated with MoS_2_-SS-HA nanosheets were higher than the control group (saline treatment) after irradiation, exhibiting that the MoS_2_-SS-HA could lead to cell apoptosis depending on the hyperthermia generated by the irradiation. Meanwhile, cells incubated with MoS_2_-SS-HA-Er were significantly less than the free Er group, verifying the stronger apoptosis effect on CD44-positive cancer cells caused by the MoS_2_-SS-HA nanosheets. Considering the hypotoxicity of MoS_2_-SS-HA nanosheets, we believed that the targeting ability played an important role in antitumor effect. Furthermore, after treated with MoS_2_-SS-HA-Er + NIR, the apoptosis rates of all cells (A549, PC-9, and H1975) were the highest among all groups, demonstrating that the combined therapy remarkably promoted the cell apoptosis. In the cell apoptosis result, the ratio of apoptosis cells in A549, PC-9 and H1975 increased to 24.6%, 36.3% and 23.6%, respectively. The apoptosis cells percentage of MoS_2_-SS-HA-Er + NIR group was significantly higher than MoS_2_-SS-HA-Er group and MoS_2_-SS-HA + NIR group, due to the synergy of chemotherapy and photothermal.

To further explore the mechanism of cell death, the result of cell cycle tested by flow cytometry was analyzed. As indicated in Fig. 6d, compared with the control group, the proportion of cells treated with MoS_2_-SS-HA + NIR in G0/G1 had no obvious difference, suggesting that the photothermal therapy alone had slight influence on the cell cycle distribution. Cells incubated with MoS_2_-SS-HA-Er showed the higher arrest percent of G0/G1 compared to the group of free Er, owing to the enhanced intracellular drug uptake mediated by the targeting of HA. After exposed under the 808 nm laser, the cells treated with MoS_2_-SS-HA-Er + NIR showed the highest ratio of G0/G1-phase because of the synergistic therapeutic effect. In summary, MoS_2_-SS-HA-Er could enhance the arrest of G0/G1 phase and prevent DNA replication, especially with 808 nm NIR irradiation, while the MoS_2_-SS-HA + NIR had no such effect. The results of the apoptosis ratio and cell cycle arrest were consistent with the conclusion of MTT assay, suggesting that the MoS_2_-SS-HA-Er + NIR induced the death of cancer cells through the G0/G1-phase arrest and cell apoptosis induction.

### Anti-tumor efficacy in vivo

**Fig. 7:**
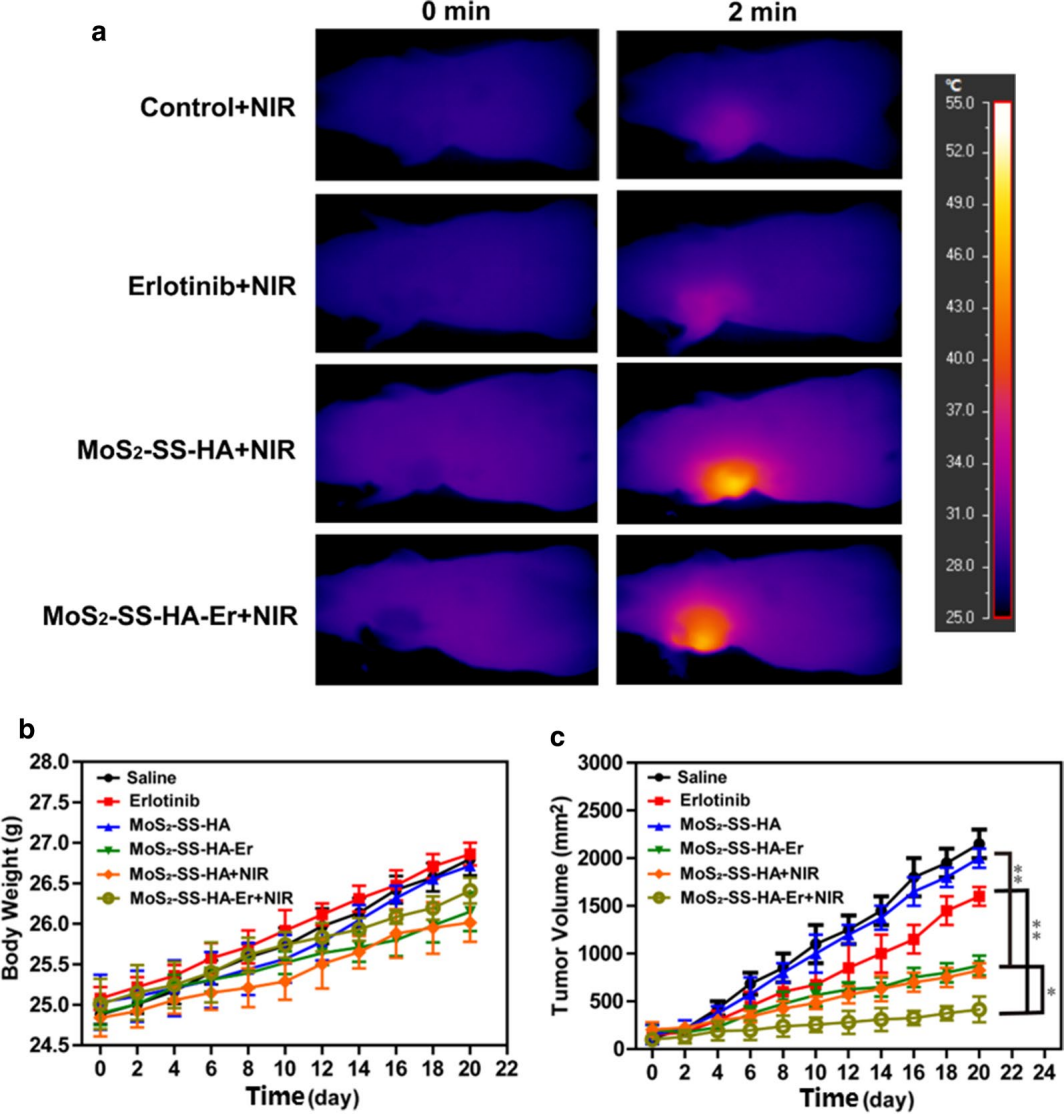
Comparative investigation of inhibiting tumor effectiveness in vivo. **a** NIR thermal images of A549 tumor-bearing mice injected with saline, Er, MoS_2_-SS-HA, and MoS_2_-SS-HA-Er + NIR. Mice body weight curves (**b**) and tumor growth curves of tumors (**c**) after various treatments for five groups. Significant differences between groups are labeled with *p < 0.05 and **p < 0.01

Encouraged by the synergistic therapeutic effect of MoS_2_-SS-HA-Er + NIR in vitro, comparative studies of inhibiting tumor effectiveness in vivo was further investigated. In the thermal images (Fig. 7a), the temperature of the tumors on the MoS_2_-SS-HA and MoS_2_-SS-HA-Er injected mice quickly increased and could readily reach a level (Δ*T *= 21 °C) which could induce hyperthermia and heat-induced drug release to kill the tumor. However, the tumor temperature of control (treated with saline) and free Er shows insignificant change (Δ*T *= 4 °C). Moreover, due to high toxicity always leading to a significant weight loss, the body weight of these mice was measured during the treatments, and no obvious weight loss was observed (Fig. 7b), indicating the low toxicity of the treatments in vivo. The tumor volumes of each group were measured and were then plotted as a function of time (Fig. 7c). Compared with the control group, efficient inhibition of tumor growth is observed for the group treated with MoS_2_-SS-HA-Er + NIR. Especially, the mean tumor volume in the MoS_2_-SS-HA-Er + NIR group is the smallest among all treated groups, which demonstrates that MoS_2_-SS-HA-Er can effectively inhibit tumor growth under the NIR laser irradiation. The reason could be attributed to (i) HA functionalized MoS_2_-SS-HA-Er targeting tumor site, and (ii) enhanced on-demand release of Er from MoS_2_-SS-HA-Er after laser irradiation, ultimately inhibiting tumor growth, further (iii) the combination of photothermal with chemotherapy therapy, both of which were activated simultaneously by 808 nm laser.

## Conclusions

In summary, a multifunctional MoS_2_-based drug delivery system (MoS_2_-SS-HA-Er) was successfully synthesized in this work and shown to allow tumor-targeting synergistic chemo-photothermal therapy. MoS_2_ nanosheets were modified with the targeting and water-soluble biomacromolecule HA to enhance biocompatibility. The nanocomposite had a uniform diameter (125 nm), and could be loaded with the insoluble anti-cancer drug Er. The release of Er is accelerated under near infrared light irradiation, which is promising for controllable drug delivery system. The nanocomposites can be specifically delivered into cancerous cells via a receptor-mediated endocytosis pathway using hyaluronic acid targeting. The nanocomposites were found to be able to induce the death of cancerous cells while leaving healthy cells unaffected. Furthermore, the MoS_2_-SS-HA-based drug delivery system can be used for synergistic cancer therapy associated with NIR-mediated hyperthermia and heat-induced local drug release in vitro and in vivo. An effective treatment of lung cancer in vivo under NIR irradiation is obtained, indicating that synergistic efficacy of hyperthermia and chemotherapy is better than hyperthermia or chemotherapy alone.

## Data Availability

All data and materials are included in the manuscript.

## Abbreviations

PTT: photothermal therapy
NIR: near-infrared
MoS_2_: molybdenum disulfide
HA: hyaluronic acid
Er: erlotinib
EGFR: epidermal growth factor receptor
TMDCs: transition metal dichalcogenides
PTA: photothermal agent
PBS: phosphate-buffered saline
TEM: transmission electron microscopy
AFM: atomic force microscopy
DLS: dynamic laser scattering
UV: UV–vis spectroscopy
FT-IR: Fourier transform infrared
EDC: ethylene dichloride
NHS: *N*-hydroxysuccinimide
RBCs: red blood cells
